# Efficacy and safety of low-dose radiotherapy in MRI-confirmed refractory chronic plantar fasciitis after extracorporeal shock wave therapy

**DOI:** 10.1016/j.ctro.2026.101236

**Published:** 2026-07-14

**Authors:** Manuel Novo-Rigueiro, Fabio Pires-Pereira, Ignacio Lete-Achirica, Antonio Gómez-Caamaño, Francisco Javier Rodríguez-Rigueiro, Jesús Rodríguez-Figueroa, Arturo González-Quintela, Ignacio Novo-Veleiro

**Affiliations:** a Department of Physical Medicine and Rehabilitation, Álvaro Cunqueiro University Hospital of Vigo, Vigo, Spain; b Department of Radiation Oncology, Clinical University Hospital of Santiago de Compostela, Santiago de Compostela, Spain; c Department of Radiology, Clinical University Hospital of Santiago de Compostela, Santiago de Compostela, Spain; d Department of Radiation Oncology, Clinical University Hospital of Santiago de Compostela, Santiago de Compostela, Spain; e Department of Engineering and Advanced Technologies, University of Santiago de Compostela (USC), Santiago de Compostela, Spain; f Department of Physical Medicine and Rehabilitation, Clinical University Hospital of Santiago de Compostela, Santiago de Compostela, Spain; g Department of Internal Medicine, Clinical University Hospital of Santiago de Compostela, Santiago de Compostela, Spain; h Department of Internal Medicine, Clinical University Hospital of Santiago de Compostela, Santiago de Compostela, Spain

**Keywords:** Plantar fasciitis, Low-dose radiotherapy, Magnetic resonance imaging, Clinical outcomes

## Abstract

**Background:**

Refractory chronic plantar fasciitis remains a therapeutic challenge when symptoms persist despite conservative management and advanced physical therapies. Although low-dose radiotherapy has been used as an anti-inflammatory treatment for benign musculoskeletal disorders, integrated data combining clinical, functional, quality-of-life and imaging outcomes remain limited.

**Methods:**

A longitudinal observational study was conducted in 68 patients with MRI-confirmed refractory chronic plantar fasciitis who remained symptomatic despite conservative treatment and extracorporeal shock wave therapy. All patients received low-dose radiotherapy (3 Gy in 6 fractions of 0.5 Gy). Pain (VAS), function (FFI), and health-related quality of life (EQ-5D) were assessed at baseline and at 1, 3, 6, 12 and 24 months. MRI was performed at baseline and at 3 and/or 6 months after treatment. Global clinical response integrated pain, function and quality-of-life outcomes.

**Results:**

Low-dose radiotherapy was associated with rapid and sustained improvement in pain, function and health-related quality of life. At 6 months, 95.6% of patients met criteria for global clinical response, which remained stable up to 24 months. Exploratory analyses showed that 85.1% of patients achieved a good clinical response during long-term follow-up, while 35.8% achieved an excellent response. Concordance between clinical and MRI response was limited. No radiation-related acute or late adverse events were identified.

**Conclusion:**

Low-dose radiotherapy was associated with sustained clinical benefit in MRI-confirmed refractory chronic plantar fasciitis after failure of conservative treatment and extracorporeal shock wave therapy. Clinical improvement frequently exceeded structural MRI changes, suggesting that symptomatic recovery may occur despite persistent imaging abnormalities.

## Highlights

• Low-dose radiotherapy provides sustained pain relief in refractory plantar fasciitis.
• Functional improvement and quality of life gains persist during long-term follow-up.
• MRI shows structural changes after radiotherapy in a subset of patients.
• Clinical response is not always paralleled by MRI morphological changes.

## 1 Introduction

Plantar fasciitis, also termed plantar fasciopathy, is among the most common causes of chronic heel pain in adults and is a frequent contributor to functional impairment and diminished quality of life. It is typically manifested as enthesopathic pain at the proximal calcaneal insertion of the plantar fascia and is linked to degenerative and inflammatory changes rather than a purely inflammatory condition [1]. Chronic plantar fasciitis is typically defined by symptoms lasting longer than six months, frequently accompanied by imaging evidence of fascial thickening and signal alteration, and by a limited or incomplete response to standard conservative treatment [1], [2].

In this context, plantar fasciitis is considered refractory when symptoms persist despite a stepwise therapeutic approach that typically includes exercise therapy, stretching programs, foot orthoses, physiotherapy, and, in many cases, additional interventional or physical treatments [2]. Although most patients experience symptom improvement with conservative measures, a relevant subgroup evolves toward refractory disease with ongoing pain and disability. For these patients, advanced therapies such as extracorporeal shock wave therapy (ESWT) are frequently employed and have demonstrated efficacy in selected patient populations [3], [4]. However, treatment response is not universal, and randomized controlled trials have reported variable clinical benefit depending on patient selection and treatment protocols [5]. Consequently, a proportion of patients continue to experience persistent symptoms despite ESWT, highlighting the need for additional therapeutic options.

Low-dose radiotherapy has long been used as an anti-inflammatory and analgesic approach for benign musculoskeletal disorders, including degenerative and enthesopathic conditions [6], [7]. Experimental and clinical evidence indicates that low doses of ionizing radiation have immunomodulatory effects, reduce inflammatory cell adhesion, and influence pain pathways, resulting in symptom relief without clinically relevant tissue adverse effects when appropriate dose regimens are used [8], [9]. More recently, an international intersociety framework has further supported the appropriate use of radiotherapy for selected benign conditions when integrated into multidisciplinary care pathways [10]. Within this context, radiotherapy has attracted renewed interest as a therapeutic option for refractory plantar fasciitis, particularly in patients who have already exhausted conservative and physical treatment approaches [11], [12], [13].

Randomized comparative studies have further strengthened the evidence supporting low-dose radiotherapy for refractory plantar fasciitis. A randomized controlled trial demonstrated superior pain relief and functional improvement with radiotherapy compared with local corticosteroid injection, whereas another randomized trial found comparable clinical outcomes between radiotherapy and platelet-rich plasma treatment in patients with chronic plantar fasciitis [14], [15].

Despite encouraging clinical evidence, important knowledge gaps remain regarding the relationship between clinical improvement, health-related quality of life, and structural MRI changes following low-dose radiotherapy. Most available studies have primarily focused on pain reduction and relatively short-term outcomes [11], [12], [13], [14], [15]. Data integrating functional improvement, health-related quality of life, and imaging findings—particularly magnetic resonance imaging (MRI)—remain scarce. In addition, the association between clinical response and MRI-detected structural changes after radiotherapy has not yet been fully elucidated.

The present study aims to evaluate the clinical effectiveness and safety of low-dose radiotherapy in a well-defined cohort of patients with MRI-confirmed refractory chronic plantar fasciitis who remained symptomatic despite comprehensive conservative management and extracorporeal shock wave therapy. By combining longitudinal assessment of pain, function, health-related quality of life, and MRI findings with extended follow-up, this study seeks to provide a comprehensive evaluation of treatment response and to better define the role of low-dose radiotherapy within the therapeutic algorithm of refractory plantar fasciitis.

## 2 Materials and methods

### 2.1 Study design and population

A longitudinal observational study was performed in patients with chronic plantar fasciitis treated at a tertiary referral hospital. An initial consecutive cohort of 309 patients with a clinical diagnosis of chronic plantar fasciitis was assessed, defined as persistent plantar heel pain for more than six months [1], [2]. 22 patients were excluded at this stage due to incomplete follow-up or insufficient clinical data.

From this initial cohort, 287 patients underwent a stepwise therapeutic protocol that included specific stretching of the plantar fascia and Achilles tendon complex, therapeutic exercise, foot orthoses, and physiotherapy. Depending on clinical evolution, some patients also received corticosteroid injections. After failure of conservative management, all patients were treated with a standardized protocol of radial extracorporeal shock wave therapy using the BTI Masterpuls® MP100 device (BTI Biotechnology Institute, Vitoria-Gasteiz, Spain). The protocol consisted of four weekly sessions delivered under direct medical supervision by the same specialist physician. Each session included 2000 impulses applied at a pressure of 3 bars and a frequency of 10 Hz over the painful plantar fascia insertion region [3], [4].

Despite this comprehensive therapeutic approach, 68 patients with MRI-confirmed plantar fasciopathy continued to experience clinically relevant pain and functional limitation, constituting a subgroup of refractory chronic plantar fasciitis. These patients were selected to receive low-dose radiotherapy and comprised the study population.

The study was conducted in accordance with the principles of the Declaration of Helsinki and was approved by the corresponding institutional ethics committee. Written informed consent was obtained from all patients prior to inclusion.

## 3 Inclusion and exclusion criteria

### 3.1 Inclusion criteria

Patients were included if they met all of the following criteria:

1. Age ≥ 18 years.
2. Plantar heel pain of at least 6 months duration, consistent with chronic plantar fasciitis.
3. Documented failure of conservative treatment, including therapeutic exercise, stretching, foot orthoses, physiotherapy, and, in some cases, corticosteroid injections.
4. Persistent clinically relevant pain leading to the indication of low-dose radiotherapy.
5. Diagnosis confirmed by MRI, performed in all patients prior to radiotherapy [13].
6. Complete clinical follow-up after treatment.

### 3.2 Exclusion criteria

Patients were excluded if they met any of the following criteria:

1. Previous surgery directly related to plantar fascia release or surgery considered responsible for the current plantar pain syndrome.
2. Concomitant conditions that could explain plantar pain (Baxter's neuropathy, stress fractures, uncontrolled adjacent tendinopathies, or other causes unrelated to plantar fasciitis) [13].
3. Contraindications to radiotherapy, including pregnancy.
4. Loss to follow-up precluding adequate clinical assessment.

### 3.3 Radiotherapy protocol

Radiotherapy was delivered in the Department of Radiation Oncology with anti-inflammatory and analgesic intent. Treatment planning was performed using a 3D simulation CT scanner (Siemens SOMATOM go.Open Pro).

**Fig. 1:**
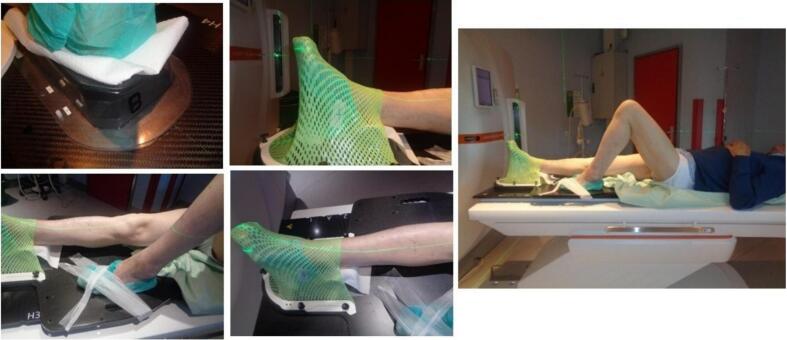
Patient positioning and immobilization during low-dose radiotherapy for plantar fasciitis. Representative positioning setup showing immobilization of the affected foot with a low-melting-point thermoplastic mask and dedicated foot support, enabling stable neutral positioning and reproducible lateral beam incidence throughout treatment delivery.

To ensure reproducible positioning, the affected foot was immobilized using a low-melting-point thermoplastic mask and dedicated foot support, allowing stable and accurate placement in a neutral position and reproducible lateral beam incidence throughout treatment delivery **(**Fig. 1**).**

Target volume delineation was performed according to international recommendations for radiotherapy in benign diseases [6], [12]:

• **Gross target volume (GTV):** lesion visible on magnetic resonance imaging.
• **Clinical target volume (CTV):** included the GTV, the proximal plantar fascia insertion, the adjacent calcaneal enthesis including the calcaneal spur when present, and the posterior calcaneal insertional region, rather than the entire Achilles tendon.
• **Planning target volume (PTV):** generated by an isotropic expansion of 1 cm from the CTV.

The prescribed dose was 3 Gy, delivered in 6 fractions of 0.5 Gy administered twice weekly, with a total treatment duration of three weeks. The dose was prescribed to the isocenter, ensuring that at least 95% of the PTV was covered by the planned isodose.

**Fig. 2:**
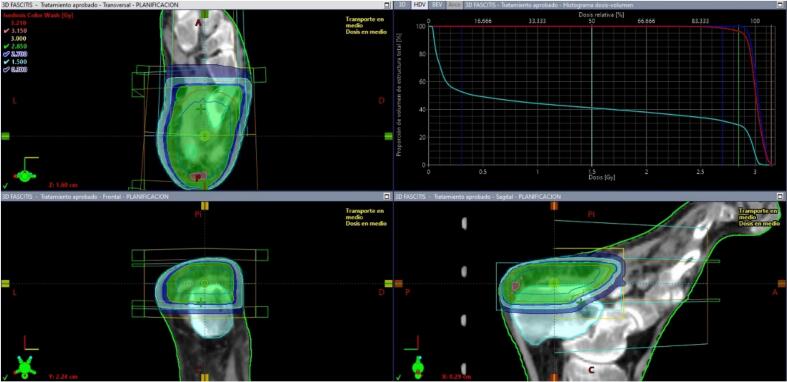
Radiotherapy planning and dose distribution for low-dose radiotherapy in plantar fasciitis. Orthogonal planning views show target volume delineation and dose distribution around the plantar fascia insertion region. The dose–volume histogram illustrates dose coverage of the clinical target volume (CTV), planning target volume (PTV), and calcaneus.

Treatment was delivered using a TrueBeam™ linear accelerator (Varian, multi-energy), employing three-dimensional conformal radiotherapy (3D-CRT) with opposed lateral fields (90°/270°) and 6 MV photons. Dose distribution and target coverage were verified using standard radiotherapy planning tools, including orthogonal dose distribution views and dose–volume histogram assessment **(**Fig. 2**).** Image-guided radiotherapy (IGRT) with daily cone-beam CT (CBCT) was used for positioning verification and correction before each fraction.

All treatments were planned and delivered by the same radiation oncologist following a standardized protocol. All prescribed fractions were completed without treatment interruption.

### 3.4 Clinical, functional and quality-of-life assessments

Patients were evaluated before the initiation of radiotherapy (baseline) and during follow-up at 1, 3, 6, 12, and 24 months after completion of treatment. All clinical and functional assessments were performed by the same specialist in Physical and Rehabilitation Medicine, ensuring intraobserver consistency.

At each evaluation, the following validated instruments were used:

• **Pain:** Visual Analogue Scale (VAS, 0–10) [16].
• **Function:** Foot Function Index (FFI), including its three domains and total score [17], [18].
• **Health-related quality of life:** EuroQol-5D questionnaire (EQ-5D), assessing its five dimensions [19].

### 3.5 Imaging assessment

All patients underwent baseline MRI before radiotherapy. Follow-up MRI was obtained in two independent patient subgroups according to the scheduled imaging assessment: 23 patients were reassessed at 3 months and a different subgroup of 45 patients at 6 months after treatment.

The analyzed variables included proximal plantar fascia thickness, presence of soft tissue edema, calcaneal bone marrow edema, and plantar fascia signal intensity [12].

All MRI studies were interpreted by the same radiologist, minimizing interobserver variability. The radiologist was not formally blinded to the timing of the examination, which was considered in the interpretation of imaging-related findings.

### 3.6 Definition of clinical response

The primary response criterion was pain improvement. A clinically meaningful pain response was defined as a reduction of ≥2 points in the VAS score compared with baseline, in accordance with established thresholds for chronic pain interventions [20].

To better characterize the magnitude of clinical benefit, additional exploratory response categories were defined:

• Minimal response: VAS reduction ≥2 points.
• Good response: VAS reduction ≥4 points or final VAS ≤ 3 points.
• Excellent response: final VAS ≤ 1 point.

Secondary response criteria included:

• Function (FFI): reduction of ≥7 points in the total score (minimal clinically important difference, MCID) [17], [18]. The total FFI score ranges from 0 to 230, with higher scores indicating greater functional impairment.
• Quality of life (EQ-5D): decrease of ≥1 level in any dimension or an increase of ≥0.05 units in the global EQ-5D index [19].
• Structural response on MRI: reduction of ≥0.5 mm in proximal plantar fascia thickness [12].
• Clinical discharge: final VAS < 5 points with concomitant functional improvement.

A global clinical responder was defined as a patient meeting the primary pain-response criterion or, alternatively, at least two secondary response criteria.

### 3.7 Safety and adverse events

Adverse events were systematically recorded during follow-up, including the occurrence of new symptoms or exacerbation of pre-existing symptoms.

### 3.8 Statistical analysis

Quantitative variables were presented as mean ± standard deviation, whereas categorical variables were summarized as absolute frequencies and percentages. Longitudinal changes between baseline and follow-up assessments were analyzed using paired statistical tests. Normality of paired differences was assessed using the Shapiro–Wilk test; paired Student's *t*-test was applied for normally distributed variables, whereas the Wilcoxon signed-rank test was used for non-normally distributed data. Associations between changes in clinical and MRI variables were explored using Spearman's rank correlation coefficient. Agreement between global clinical response and MRI-defined response was assessed using Cohen's kappa coefficient. Exploratory univariate logistic regression analyses were performed to identify baseline predictors of global clinical response. A two-sided *p* value <0.05 was considered statistically significant. Statistical analyses were performed using IBM SPSS Statistics version 29.0 (IBM Corp., Armonk, NY, USA).

## 4 Results

### 4.1 Patient characteristics

A total of 68 patients with refractory chronic plantar fasciitis were included in the analysis. Baseline demographic and clinical characteristics are summarized in Table 1. The mean age was 52.6 ± 11.2 years, and most patients were female (72.1%). A physically demanding occupation was reported by 77.6% of patients. Relevant comorbidities included hypothyroidism (27.9%), diabetes mellitus (17.6%), and hypertension (17.6%). Nearly half of the patients (48.5%) had received prior corticosteroid injections. Baseline pain and functional impairment were severe, with a mean VAS score of 8.38 ± 0.65 and a mean Foot Function Index (FFI) score of 194.75 ± 19.64.

**Table 1:** Baseline characteristics of patients treated with low-dose radiotherapy.

Variable	Value
Age, years (mean ± SD)	52.6 ± 11.2
Female sex, n (%)	49 (72.1%)
Physically demanding occupation, n (%)	52 (77.6%)
Bilateral plantar fasciitis, n (%)	0 (0.0%)
Diabetes mellitus, n (%)	12 (17.6%)
Hypothyroidism, n (%)	19 (27.9%)
Hypertension, n (%)	12 (17.6%)
Fibromyalgia, n (%)	6 (8.8%)
Rheumatologic disease, n (%)	2 (2.9%)
Smoker, n (%)	11 (16.2%)
Alcohol consumption, n (%)	4 (5.9%)
Prior foot surgery, n (%)	3 (4.4%)
Prior corticosteroid injection, n (%)	33 (48.5%)
Baseline pain (VAS), mean ± SD	8.38 ± 0.65
Baseline Foot Function Index (FFI), mean ± SD	194.75 ± 19.64
Baseline EQ-5D mobility ≥ level 2, n (%)	68 (100.0%)

Demographic data, comorbidities, prior treatments and baseline clinical status of the 68 patients with chronic refractory plantar fasciitis included in the study. Pain intensity was assessed using VAS, FFI, and health-related quality of life using the EQ-5D questionnaire.

### 4.2 Pain and functional outcomes

**Fig. 3:**
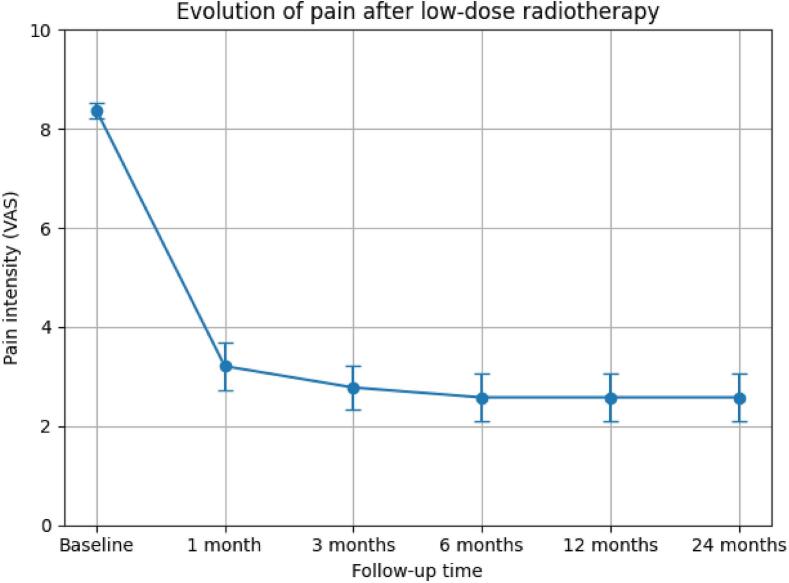
Evolution of pain intensity (VAS) after low-dose radiotherapy. Mean pain intensity measured using VAS (0−10) at baseline and during follow-up at 1, 3, 6, 12 and 24 months after low-dose radiotherapy. Data are presented as mean values with 95% confidence intervals. A marked reduction in pain was observed as early as 1 month, with sustained improvement throughout long-term follow-up.

A marked and sustained reduction in pain intensity was observed after low-dose radiotherapy. Mean VAS scores decreased significantly from baseline to the first month of follow-up and continued to improve over time **(**Fig. 3**; Supplementary Table 1)**. Pain reduction remained stable at 6, 12, and 24 months after treatment.

**Fig. 4:**
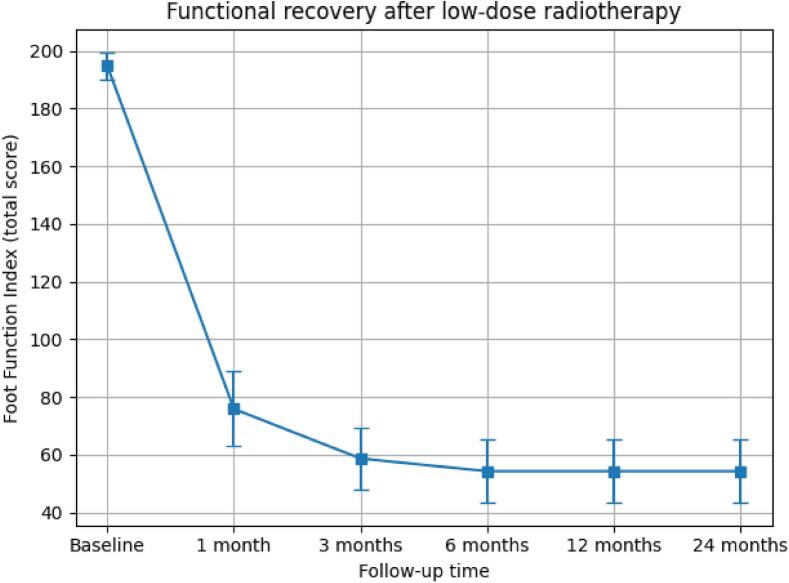
Functional recovery after low-dose radiotherapy. Mean total FFI scores at baseline and during follow-up at 1, 3, 6, 12 and 24 months after treatment. Data are presented as mean values with 95% confidence intervals. A marked functional improvement was observed within the first month after radiotherapy, with further gains at 3 months and sustained recovery throughout long-term follow-up.

Functional outcomes showed a parallel improvement. Mean FFI scores, where higher values indicate greater functional impairment, decreased substantially from baseline to follow-up assessments, with clinically meaningful improvements observed as early as the first month and maintained throughout the entire follow-up period **(**Fig. 4**; Supplementary Table 2)**.

### 4.3 Health-related quality of life

**Fig. 5:**
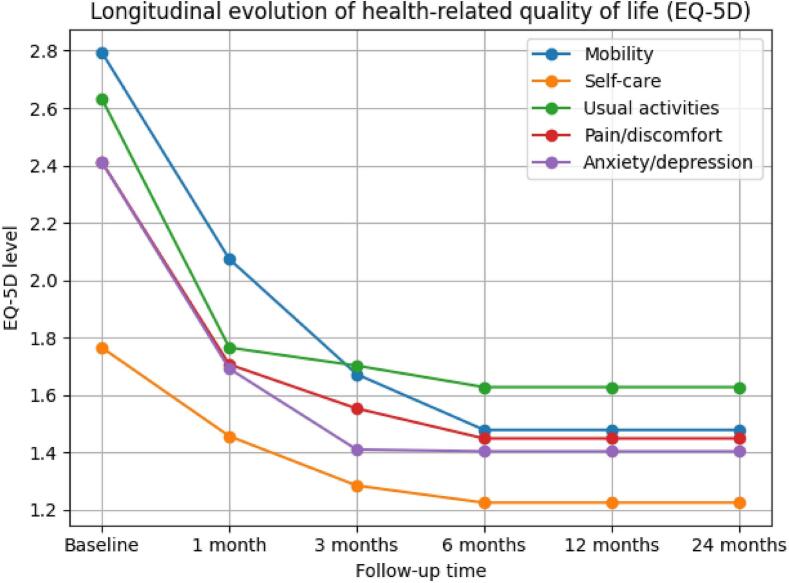
Longitudinal evolution of health-related quality of life assessed by EQ-5D. Mean EQ-5D levels by dimension (mobility, self-care, usual activities, pain/discomfort and anxiety/depression) from baseline to 24 months after low-dose radiotherapy. Lower scores indicate better health status. An early improvement was observed across all dimensions at 1 month, with further gains up to 6 months and sustained benefits during long-term follow-up.

Significant improvements were observed across all EQ-5D dimensions following radiotherapy. Mean scores for mobility, self-care, usual activities, pain/discomfort, and anxiety/depression progressively improved from baseline to follow-up, with the greatest changes observed within the first 6 months after treatment **(**Fig. 5**; Supplementary Table 3)**. These improvements remained stable during longer-term follow-up.

### 4.4 Clinical response rates

Clinical response rates were consistently high across all evaluated clinical domains. At 6 months, 95.6% of patients met response criteria for pain improvement, 98.5% showed clinically meaningful functional improvement, and 92.6% demonstrated improvement in health-related quality of life. When applying the predefined composite global response criterion integrating pain, function, and quality of life, 95.6% of patients were classified as global clinical responders (Table 2).

**Table 2:** Clinical response by outcome domain at 6 months after low-dose radiotherapy.

Outcome domain	Responders (n/N)	Responders (%)
Pain (VAS)	65/68	95.6
Function (FFI)	67/68	98.5
Quality of life (EQ-5D)	63/68	92.6
Global composite response*	65/68	95.6

Proportion of responders according to pain intensity (VAS), functional outcome (FFI), health-related quality of life (EQ-5D), and global clinical response. Response criteria were predefined for each outcome measure. *Global clinical response was defined as fulfillment of the primary pain-response criterion or, alternatively, at least two secondary response criteria.

Additional exploratory response categories were analyzed to better characterize the magnitude of pain improvement after radiotherapy. At 3 months, 91.2% of patients achieved a good clinical response, defined as a VAS reduction ≥4 points or a final VAS ≤3 points, while 27.9% achieved an excellent response, defined as a final VAS ≤1 point. Sustained good response rates remained stable during long-term follow-up, reaching 85.1% at 6, 12, and 24 months. Excellent response rates increased over time, reaching 35.8% during long-term follow-up.

### 4.5 MRI findings and concordance with clinical response

All patients underwent baseline MRI evaluation. Follow-up MRI was available in two independent patient subgroups: 23 patients were reassessed at 3 months and a different subgroup of 45 patients at 6 months after radiotherapy. Structural changes on MRI included variations in proximal plantar fascia thickness, soft tissue edema, calcaneal bone marrow edema, and plantar fascia signal intensity.

The concordance between MRI-based response and global clinical response was limited. Cohen's kappa values ranged from 0.047 to 0.281 at 3 months and from −0.087 to 0.051 at 6 months, indicating poor agreement between imaging findings and clinical improvement (Table 3).

**Table 3:** Clinical–radiological concordance between global clinical response and MRI findings.

Parameter	Kappa (3 months)	n (3 months)	Kappa (6 months)	n (6 months)
Thickness	0.047	23	0.051	45
Soft tissue edema	0.057	23	−0.087	45
Bone marrow edema	0.112	23	−0.081	45
Signal intensity	0.281	23	0.051	45

Agreement between global clinical response and changes in magnetic resonance imaging (MRI) parameters at 3 and 6 months after low-dose radiotherapy, assessed using Cohen's kappa coefficient. At 3 months, reduction in signal intensity showed the highest concordance with clinical response, whereas other structural and inflammatory parameters demonstrated limited agreement. At 6 months, concordance remained low across MRI parameters, likely reflecting the very high prevalence of clinical response and the heterogeneous temporal resolution of radiological findings. The 3-month (*n* = 23) and 6-month (*n* = 45) MRI analyses were performed in two independent follow-up cohorts.

### 4.6 Predictors of clinical response

Exploratory univariate analyses were performed only as hypothesis-generating analyses because of the very small number of non-responders. No consistent baseline predictors of global clinical response were identified. Although fibromyalgia was associated with a lower probability of achieving a good clinical response in exploratory analyses, this finding should be interpreted with particular caution and requires confirmation in larger cohorts **(Supplementary Table 4)**.

### 4.7 Safety

Low-dose radiotherapy was well tolerated. Only one patient reported worsening of pre-existing bilateral foot paresthesias and increased episodes of epistaxis during follow-up. Both symptoms had been present before radiotherapy and were not considered treatment-related by the clinical team. No acute or late radiation-related adverse events were identified during follow-up.

## 5 Discussion

The present study indicates that low-dose radiotherapy is associated with substantial, clinically meaningful, and durable improvement in a particularly difficult-to-treat cohort of patients with MRI-confirmed refractory chronic plantar fasciitis who remained symptomatic despite an extensive stepwise treatment strategy, including conservative measures and extracorporeal shock wave therapy [3], [4]. Improvements were observed in pain, function, and health-related quality of life, with high global clinical response rates sustained throughout long-term follow-up. These findings support the potential role of low-dose radiotherapy as a salvage therapeutic option in selected refractory cases.

Pain relief was rapid and substantial, with a significant improvement already evident at 1 month after treatment and maintained through 24 months. This pattern is consistent with prior reports on the analgesic effects of low-dose radiotherapy in benign musculoskeletal disorders and enthesopathies [6], [9], [10], [11]. The extent and persistence of pain relief are especially noteworthy in this refractory cohort, in which conventional conservative management and advanced physical therapies had already been unsuccessful.

Functional outcomes, measured with the FFI, improved in parallel and in a clinically meaningful manner. Functional recovery is a key endpoint in plantar fasciitis, as pain-related disability frequently translates into restrictions in daily activities and work performance [17], [18]. The rapid and durable reduction in FFI scores seen in this cohort indicates that radiotherapy has clinical value beyond analgesia and supports its potential contribution to functional restoration in chronic, refractory disease.

Health-related quality of life also improved significantly across all EQ-5D dimensions. Improvements in mobility, self-care, usual activities, pain/discomfort, and anxiety/depression highlight the broader impact of symptom relief on patient well-being [19]. These findings underscore the importance of incorporating patient-reported outcome measures when evaluating therapeutic interventions in chronic musculoskeletal conditions, particularly in benign diseases where treatment goals extend beyond symptom control to overall quality of life.

Despite the marked clinical response, agreement between clinical improvement and structural MRI changes was low. Poor concordance was reflected by Cohen's kappa values comparing global clinical response with MRI-derived parameters, including plantar fascia thickness, edema, and signal intensity. Although MRI showed improvement in selected structural and inflammatory-related findings in a subset of patients, several individuals achieved marked clinical improvement despite persistent imaging abnormalities. This apparent dissociation is consistent with previous studies showing that plantar fascia thickness does not necessarily correlate with pain intensity, functional impairment, or quality of life in patients with recalcitrant plantar fasciitis [21]. Therefore, persistence of selected structural MRI abnormalities should not necessarily be interpreted as ongoing clinically relevant disease. Inflammatory MRI findings, including soft tissue edema, may also resolve at a different pace than patient-reported symptoms. These findings support the concept that the primary therapeutic goal in refractory plantar fasciitis should remain clinical and functional improvement rather than complete radiological normalization. This mismatch may indicate that symptom improvement following low-dose radiotherapy is driven mainly by anti-inflammatory and neuromodulatory effects, rather than by early or measurable structural remodeling of the plantar fascia [8], [9], [13].

The exploratory analysis of baseline predictors was intentionally limited to hypothesis generation because of the very small number of non-responders. No consistent demographic, clinical, or treatment-related factors were identified as reliable predictors of global clinical response. Although fibromyalgia was associated with a lower probability of achieving a good clinical response in univariate analysis, this observation should be considered preliminary and interpreted with particular caution. Larger studies are required before any firm conclusions can be drawn regarding predictors of response or patient selection.

From a safety perspective, low-dose radiotherapy was well tolerated. During follow-up, only one patient experienced an exacerbation of pre-existing symptoms, and there were no new or serious treatment-related adverse events. This aligns with the documented safety profile of low-dose radiotherapy in benign disease when suitable dosing schedules and contemporary radiation techniques are applied [9], [10], [11], [12], [13].

Several limitations should be acknowledged. First, this was a single-center observational study without a control group, in particular, the absence of a sham or comparator group precludes exclusion of potential placebo-related effects; which limits causal inference regarding treatment efficacy. Second, although the cohort was prospectively followed, the absence of randomization may have introduced selection bias. Third, follow-up MRI examinations were available only for a subgroup of patients, reducing the statistical power of imaging-based analyses. In addition, MRI assessments were not formally blinded to examination timing, which may have introduced interpretation bias. Finally, exploratory analyses of predictors of response should be interpreted cautiously due to the small number of non-responders. Several aspects strengthen the present study. The cohort included only patients with MRI-confirmed refractory plantar fasciitis after failure of conservative management and extracorporeal shock wave therapy, representing a highly selected difficult-to-treat population. In addition, all radiotherapy treatments were planned and delivered using a standardized protocol by the same radiation oncologist, while clinical and imaging assessments were consistently performed by the same rehabilitation physician and radiologist, respectively. This methodological consistency likely reduced interobserver variability and improved the reproducibility of the longitudinal assessments.

In conclusion, this study supports low-dose radiotherapy as a potential therapeutic option for carefully selected patients with MRI-confirmed refractory chronic plantar fasciitis who remain symptomatic after conservative management and extracorporeal shock wave therapy. The sustained improvements in pain, function, and health-related quality of life, together with the favorable safety profile observed during follow-up, reinforce the clinical value of this approach in a difficult-to-treat population. The limited agreement between clinical and MRI response further suggests that treatment success in this setting should be primarily judged by patient-centered clinical outcomes rather than by complete structural normalization on imaging.

## 6 Conclusions

Low-dose radiotherapy was associated with significant and sustained clinical benefit in patients with refractory chronic plantar fasciitis who had failed a comprehensive stepwise therapeutic approach, including conservative management and extracorporeal shock wave therapy.

In this well-characterized cohort, marked pain reduction, clinically meaningful functional improvement, and relevant enhancement in health-related quality of life were observed after radiotherapy, with high rates of global clinical response maintained over long-term follow-up. These benefits occurred despite limited agreement between clinical improvement and structural changes on magnetic resonance imaging, suggesting that the therapeutic effect of low-dose radiotherapy may be mediated predominantly by anti-inflammatory and neuromodulatory mechanisms rather than by early structural remodeling.

Low-dose radiotherapy showed a favorable safety profile, with no serious treatment-related adverse events observed during follow-up, supporting its use in benign musculoskeletal conditions when modern techniques and appropriate dose regimens are employed.

Overall, these findings support low-dose radiotherapy as a potential therapeutic option for patients with refractory chronic plantar fasciitis and reinforce its possible role within the therapeutic algorithm for this challenging condition.

## AI declaration

As non-native English speakers, the authors of this study employed artificial intelligence tools to improve its clarity and readability. The manuscript content was reviewed and edited by the authors before submission, acknowledging full responsibility for the accuracy and integrity of the work.

## CRediT authorship contribution statement

**Manuel Novo-Rigueiro:** Conceptualization, Methodology, Investigation, Data curation, Formal analysis, Writing – original draft, Writing – review & editing, Supervision. **Fabio Pires-Pereira:** Investigation, Data curation, Writing – review & editing. **Ignacio Lete-Achirica:** Investigation, Data curation, Writing – review & editing. **Antonio Gómez-Caamaño:** Conceptualization, Methodology, Writing – review & editing. **Francisco Javier Rodríguez-Rigueiro:** Formal analysis, Writing – review & editing. **Jesús Rodríguez-Figueroa:** Investigation, Writing – review & editing. **Arturo González-Quintela:** Conceptualization, Writing – review & editing. **Ignacio Novo-Veleiro:** Conceptualization, Supervision, Writing – review & editing.

## Ethics approval and consent to participate

A prospective observational study was conducted at the University Hospital Complex of Santiago de Compostela and within the Santiago–Barbanza health area between January 2019 and December 2025. The study protocol was approved by the Galician Research Ethics Committee (approval code 2021/120). All participants provided written informed consent prior to inclusion, in accordance with the Declaration of Helsinki and European biomedical research regulations.

## Funding

This study did not receive any specific external funding.

## Declaration of competing interest

The authors declare no conflicts of interest related to this study.

## Data Availability

The datasets generated and analyzed during the current study are available from the corresponding author on reasonable request.
