# Supplementary material for: Efficacy and safety of low-dose radiotherapy in MRI-confirmed refractory chronic plantar fasciitis after extracorporeal shock wave therapy
Source: Clin Transl Radiat Oncol. 2026 Jul 14;60:101236. doi: 10.1016/j.ctro.2026.101236 (PMC13400277; doi:10.1016/j.ctro.2026.101236)
Supplement: Supplementary material 1 [file mmc1.docx]

**Supplementary Table 1. Longitudinal clinical VAS after low-dose radiotherapy.**

| **Timepoint** | **n** | **Mean VAS** | **SD** |
| --- | --- | --- | --- |
| Baseline | 68 | 8.38 | 0.65 |
| 1 month | 68 | 3.21 | 2.03 |
| 3 months | 68 | 2.78 | 1.84 |
| 6 months | 67 | 2.58 | 2.05 |
| 12 months | 67 | 2.58 | 2.05 |
| 24 months | 67 | 2.58 | 2.05 |

Mean values and standard deviations (SD) of pain intensity assessed by the Visual Analog Scale (VAS) at baseline and during follow-up, showing a rapid and sustained improvement after treatment.
