# Supplementary material for: Efficacy and safety of low-dose radiotherapy in MRI-confirmed refractory chronic plantar fasciitis after extracorporeal shock wave therapy
Source: Clin Transl Radiat Oncol. 2026 Jul 14;60:101236. doi: 10.1016/j.ctro.2026.101236 (PMC13400277; doi:10.1016/j.ctro.2026.101236)
Supplement: Supplementary material 2 [file mmc2.docx]

**Supplementary Table 2. Longitudinal clinical FFI Total after low-dose radiotherapy.**

| **Timepoint** | **n** | **Mean FFI** | **SD** |
| --- | --- | --- | --- |
| Baseline | 68 | 194.75 | 19.64 |
| 1 month | 68 | 75.87 | 54.32 |
| 3 months | 68 | 58.69 | 45.65 |
| 6 months | 67 | 54.30 | 45.87 |
| 12 months | 67 | 54.30 | 45.87 |
| 24 months | 67 | 54.30 | 45.87 |

Mean values and standard deviations (SD) of functional impairment assessed by FFI at baseline and during follow-up. Both pain and functional scores showed a rapid and sustained improvement following treatment.
