# Supplementary material for: Efficacy and safety of low-dose radiotherapy in MRI-confirmed refractory chronic plantar fasciitis after extracorporeal shock wave therapy
Source: Clin Transl Radiat Oncol. 2026 Jul 14;60:101236. doi: 10.1016/j.ctro.2026.101236 (PMC13400277; doi:10.1016/j.ctro.2026.101236)
Supplement: Supplementary material 3 [file mmc3.docx]

**Supplementary Table 3. Longitudinal evolution of health-related quality of life assessed by EQ-5D.**

| **EQ-5D dimension** | **Baseline** | **1 month** | **3 months** | **6 months** | **12 months** | **24 months** |
| --- | --- | --- | --- | --- | --- | --- |
| Mobility | 2.79 ± 0.41 | 2.07 ± 0.47 | 1.67 ± 0.66 | 1.48 ± 0.64 | 1.48 ± 0.64 | 1.48 ± 0.64 |
| Self-care | 1.76 ± 0.65 | 1.46 ± 0.53 | 1.28 ± 0.49 | 1.22 ± 0.42 | 1.22 ± 0.42 | 1.22 ± 0.42 |
| Usual activities | 2.63 ± 0.52 | 1.76 ± 0.55 | 1.70 ± 0.60 | 1.63 ± 0.67 | 1.63 ± 0.67 | 1.63 ± 0.67 |
| Pain/discomfort | 2.41 ± 0.60 | 1.71 ± 0.62 | 1.55 ± 0.68 | 1.45 ± 0.68 | 1.45 ± 0.68 | 1.45 ± 0.68 |
| Anxiety/depression | 2.41 ± 0.67 | 1.69 ± 0.60 | 1.41 ± 0.63 | 1.40 ± 0.68 | 1.40 ± 0.68 | 1.40 ± 0.68 |

Mean EQ-5D levels by dimension at baseline and during follow-up at 1, 3, 6, 12 and 24 months after low-dose radiotherapy. A consistent improvement was observed across all dimensions, with early changes at 1 month and sustained benefits throughout long-term follow-up.
