# Supplementary material for: Efficacy and safety of low-dose radiotherapy in MRI-confirmed refractory chronic plantar fasciitis after extracorporeal shock wave therapy
Source: Clin Transl Radiat Oncol. 2026 Jul 14;60:101236. doi: 10.1016/j.ctro.2026.101236 (PMC13400277; doi:10.1016/j.ctro.2026.101236)
Supplement: Supplementary material 4 [file mmc4.docx]

**Supplementary Table 4. Baseline characteristics and exploratory univariate analysis according to global clinical response.**

| **Variable** | **Responders** | **Non-responders** | **Odds ratio** | **p-value** |
| --- | --- | --- | --- | --- |
| Age, years (mean) | 52.9 | 47.7 | 1.02 | 0.713 |
| Female sex, % | 72.3 | 66.7 | 1.31 | 1.000 |
| Physically demanding occupation, % | 78.1 | 0.0 | 0.00 | 1.000 |
| Diabetes mellitus, % | 17.2 | 33.3 | 0.41 | 0.447 |
| Hypothyroidism, % | 26.6 | 33.3 | 0.77 | 1.000 |
| Hypertension, % | 17.2 | 33.3 | 0.41 | 0.447 |
| Fibromyalgia, % | 7.8 | 33.3 | 0.17 | 0.245 |
| Prior corticosteroid injection, % | 46.9 | 66.7 | 0.46 | 0.608 |

Comparison of baseline demographic and clinical variables between responders and non-responders according to the composite global clinical response at 6 months after low-dose radiotherapy. Odds ratios and p-values were obtained using univariate logistic regression for continuous variables and Fisher’s exact test for categorical variables. Confidence intervals were omitted due to the very small number of non-responders, and results should therefore be interpreted as exploratory.
